# Feasibility of fNIRS for assessing n-back task-related prefrontal cortex oxygenation after continuous cycling at varying intensities: an evaluation of protocol feasibility

**DOI:** 10.3389/fnhum.2026.1856059

**Published:** 2026-07-31

**Authors:** Julius Debertshaeuser, Olga Tarassova, Jana Strahler, Sara Klinger, Emerald G. Heiland, Maria M. Ekblom

**Affiliations:** 1 Department of Sportspsychology, Department of Sport and Sport Science, Albert-Ludwigs-University Freiburg, Freiburg, Germany; 2 Department of Physical Activity and Health, The Swedish School of Sport and Health Sciences (GIH), Stockholm, Sweden; 3 Department of Physiology, Nutrition and Biomechanics, The Swedish School of Sport and Health Sciences (GIH), Stockholm, Sweden; 4 Department of Surgical Sciences, Medical Epidemiology, Uppsala University, Uppsala, Sweden; 5 Division of Physiotherapy, Department of Neurobiology, Care Sciences and Society, Karolinska Institutet, Huddinge, Sweden

**Keywords:** acute exercise, feasibility, fNIRS, QT-NIRS, scalp coupling index

## Abstract

**Introduction:**

While previous research has shown that single bouts of acute exercise can enhance cognitive performance, the physiological mechanisms behind this remain unclear. Functional near-infrared spectroscopy (fNIRS) constitutes a promising tool to capture brain activity in this research field, but the feasibility of implementing such setups in combination with stationary cycling has not yet been evaluated. Thus, in this exploratory feasibility and methodological validation study, we investigated (1) procedural feasibility of the experimental protocol and (2) scientific feasibility, including dual-metric data quality assessment via scalp coupling index (SCI) and quality testing of near-infrared scans (QT-NIRS), as well as behavioral and prefrontal hemodynamic indicators of n-back task engagement.

**Methods:**

Of 14 eligible, nine young and healthy participants (2 female, 7 male) completed 20 min of low-, moderate- and high-intensity continuous-cycling in a randomized cross-over design. Prefrontal cortex (PFC) activation was captured during numerical n-back tasks, administered before and with a 10-min delay after exercise. Procedural feasibility was descriptively assessed via participant attrition, protocol timing adherence, fNIRS setup reliability, target exercise load estimation, and monitoring of affect and fatigue. Scientific feasibility was evaluated through average SCI values, agreement between oxygenated and deoxygenated hemoglobin (oxyHb, deoxyHb) *β*-coefficients derived from SCI- and QT-NIRS-based pruning (Lin’s concordance correlation coefficient, CCC), and n-back task sensitivity via accuracy, reaction time, and load-dependent prefrontal hemodynamic responses.

**Results:**

Technical difficulties (*n* = 1), physical discomfort due to cap wear (*n* = 2), and undisclosed reasons (*n* = 2) were reasons for study discontinuation. Protocol timing adherence and fNIRS setup reliability were satisfactory overall, and target exercise load estimation was successful after manual adaptation. Affect and fatigue were stable across timepoints. The channel-averaged SCI value of 0.964 (*SE* = 0.003) and near-optimal *β*-agreement between techniques (oxyHb CCC = 0.958; deoxyHb CCC = 0.983) suggested that good quality data were retrieved from the setup. N-back task behavioral and hemodynamic outcomes were load-dependent.

**Discussion:**

These findings provide preliminary support for the scientific feasibility of assessing n-back task-induced changes in PFC activation using fNIRS. Future studies should consider minor adjustments to pre-experimental design and fitness assessment to potentially reduce attrition and improve target exercise load estimation.

## 1 Introduction

Evidence suggesting that acute physical exercise can influence cognitive performance has been available for more than half a century (Davey, 1973). Decades of subsequent research have substantiated this finding, with meta-analytical evidence suggesting a small positive effect of a single bout of exercise on cognitive performance (Lambourne and Tomporowski, 2010; Chang et al., 2012). Despite consistent behavioral findings, the neural correlates underlying these acute effects remain unclear.

Acute exercise may affect different domains of executive functions, and these effects likely depend on exercise intensity and assessment timing: The *prefrontal cortex* (PFC) is critically involved in human executive functions (Fuster, 2001; Koechlin and Summerfield, 2007), which encompass core top-down cognitive processes necessary for goal-directed behavior (Diamond, 2013). Although neuroimaging studies have consistently demonstrated that PFC activity is altered in response to physical exercise (Moriarty et al., 2019; Yanagisawa et al., 2010; Park and Schott, 2022), domain-specificity is illustrated by Tempest et al. (2017). The authors reported that a 60-min bout of vigorous cycling improved inhibitory control but impaired updating under parametrically changing load (as measured by the 2-back task) compared with very low-intensity cycling when cognitive tests were administered during exercise. This is supported by numerous studies that have observed increased PFC activity to coincide with reduced reaction time during inhibitory control tasks (Yanagisawa et al., 2010; Byun et al., 2014b; Kujach et al., 2018; Damrongthai et al., 2021), whereas evidence linking exercise to updating-related PFC activation is scarce and inconsistent (Bediz et al., 2016; Stute et al., 2020). Notably, recent working-memory theory emphasizes that *“working memory”* (WM) is an umbrella construct encompassing multiple subfunctions (Adam et al., 2025) – where the capacity of updating under changing load is validly captured by the n-back task but insufficient as an exhaustive measure of WM (Burgoyne et al., 2025).

Domain-specificity is further complicated by the transient and intensity-dependent nature of cognitive effects (Chang et al., 2012), combined with substantial heterogeneity in when assessments are conducted across studies – resulting in compromised interpretability. Consequently, detecting domain-specific and sometimes opposing effects within narrow temporal windows places high demands on the sensitivity and reliability of neuroimaging measures.

*Functional near-infrared spectroscopy* (fNIRS) constitutes a promising non-invasive tool to indirectly assess cortical activation by monitoring oxygenation changes (Scholkmann et al., 2014; Herold et al., 2018) and is technically well-suited for use before, during, and after exercise due to its relative robustness to motion artifacts (Yücel et al., 2017; Herold et al., 2018). However, the feasibility of applying fNIRS in exercise-cognition research depends on whether high-quality data can be obtained without adding excessive burden on the participants, which itself could introduce cognitive or hemodynamic confounds.

fNIRS signals are susceptible to systemic physiological influences and motion-related noise, necessitating careful pre-processing and quality control. The rapid growth of fNIRS research has introduced challenges related to interpretability and reproducibility, driven in part by substantial variability in pre-processing and statistical approaches (Pfeifer et al., 2018; Klein and Kranczioch, 2019). This variability necessitates rigorous signal quality control to secure reproducibility. Yücel et al. (2025) demonstrated this compellingly: Participants with higher signal-to-noise ratios showed markedly greater agreement across analytical approaches compared to those with poorer signal quality.

A practical trade-off emerges: Achieving high signal quality through recalibration, and extended baseline and setup periods conflicts with lengthy experimental sessions that may impose physical discomfort on participants (Kassab et al., 2015; Baker et al., 2017). This discomfort can be of particular concern since affective state (Pessoa, 2009) and mental fatigue (Van Der Linden et al., 2003) have been shown to modulate prefrontal-dependent cognition and potentially confound task-related measurements. Managing good signal quality in the face of minimized session duration is particularly challenging. Consequently, it remains unclear whether both *procedural* and *scientific* feasibility can be achieved when applying fNIRS to capture task-related PFC activation in close temporal proximity to exercise cessation.

Therefore, the main objective of this study is to evaluate the procedural and scientific feasibility of capturing changes in n-back task-related PFC activation before and immediately after exercise. As no *a priori* feasibility thresholds were defined, all feasibility assessments are exploratory and descriptive. Procedural feasibility assessments include participant attrition, protocol timing adherence, fNIRS setup reliability, target exercise load estimation, and monitoring of affect and fatigue throughout the experiment as potential modulators of cognitive performance. Scientific feasibility is assessed using established data-quality metrics, including the *Scalp Coupling Index* (SCI). Exploratory sensitivity analyses will be conducted using the novel *Quality Testing of Near-Infrared Scans* (QT-NIRS) pruning approach (Hernandez and Pollonini, 2020; Pollonini et al., 2014, 2016), which has received support in recent overview papers (Ayaz et al., 2022; Ivanova et al., 2026) for its nuanced signal quality assessment. Unlike SCI, which evaluates overall optode-scalp coupling, QT-NIRS additionally incorporates *peak spectral power* (PSP) to identify motion-contaminated windows, potentially enabling more targeted channel rejection. As the choice of pruning method directly affects the number of retained channels and downstream brain activation estimates, comparing both approaches provides insight into the robustness and reproducibility of fNIRS preprocessing pipelines in exercise settings. To the best of our knowledge, this is the first study directly comparing these two approaches. Additionally, to verify task engagement, behavioral and prefrontal hemodynamic load effects of the n-back task were assessed.

Evaluating the feasibility of the present study protocol, data processing, and analysis steps is intended to inform the design of future exercise-cognition studies employing time-sensitive neuroimaging assessments.

## 2 Materials and methods

### 2.1 Ethical approval

This study was approved by the Swedish Ethical Review Authority (Dnr 2022–07297-01) and conducted in accordance with the Declaration of Helsinki. At the initial meeting, all recruited participants received detailed study information and signed written informed consent prior to participation in the study.

### 2.2 Study design

This three-condition randomized-crossover-design-study encompassed a total of four attendance sessions, all of which took place at the Swedish School of Sport and Health Sciences (GIH) in Stockholm, Sweden. The initial on-site visit included three sub-sessions: (1) screening of participation criteria using a battery of questionnaires, (2) fitness assessment and (3) familiarization. Only participants who met the inclusion criteria, assessed directly after the screening, were invited to participate in the fitness assessment and familiarization. The initial visit was followed by the three experimental sessions of low-, moderate-, and high-intensity cycling, conducted on separate days in a randomized order, with a minimum 4-day washout period between sessions.

### 2.3 Participants

Twenty-one young adults from the area surrounding Stockholm, Sweden, were recruited for the study between March and June 2023. The recruitment was carried out through online advertisement and verbal promotion during university classes at the Swedish School of Sports and Health Sciences. As the aim of this pilot study was solely to ensure scientific and procedural feasibility, no *a priori* power analysis was conducted.

We included 14 prospects within the age range of 20–40 years. The exclusion criteria comprised: (1) high self-rated stress-related symptoms (Lundgren-Nilsson et al., 2012); (2) symptoms of dementia (Palmqvist et al., 2013); (3) symptoms of alcohol use disorder (Berman, 2012); (4) symptoms of anxiety (Lisspers et al., 1997) and (5) symptoms of depression (Lisspers et al., 1997). Further exclusion criteria were screened for in a health declaration survey and encompassed smoking, post-COVID symptoms, chronic medication known to alter cognition, chronic diseases, and symptoms such as high blood pressure, COPD, migraine, and bipolar disorder, somatic diseases, and musculoskeletal injuries interfering with the physical exercise introduced in the study. Health declaration responses were based on self-report without independent verification. Ambiguous cases were resolved in consultation with a licensed physician. Eligibility screening was conducted exclusively in Swedish. Table 1 lists exclusion specifications.

**Table 1:** Overview of screening criteria and exclusions.

Parameter	Instrument	Exclusion threshold	Administrator	Number of exclusions
Stress	Shirom-Melamed Burnout Questionnaire (SMBQ)	>3.75	Investigator	5
Dementia	Mini-Mental State Examination Swedish Version (MMSE-SR)	<26	Investigator	0
Alcohol use disorder	Alcohol Use Disorder Identification Test (AUDIT)	Female >6 Male >8	Investigator	2
Anxiety	Hospital Anxiety and Depression Scale (HAD)	>10	Investigator	3
Depression	Hospital Anxiety and Depression Scale (HAD)	>10	Investigator	1
Health declaration	Various	Investigator judgment	Self-report	1

Participant attrition constitutes a feasibility-relevant outcome and is therefore addressed in more detail in results.

### 2.4 Fitness assessment

Participants’ cardiorespiratory fitness was estimated utilizing the submaximal Ekblom-Bak test (Ekblom-Bak et al., 2014) on a stationary Monark 828 E (Monark Exercise AB, Vansbro, Sweden). Since this test is known to overestimate the maximal oxygen uptake (V̇O2_max_) in low-fitness populations (Björkman et al., 2016) and participants exhibited signs of exhaustion, an experienced exercise physiologist re-estimated individual fitness levels based on clinical judgment. Target workloads for each intensity condition were subsequently calculated from the *American College of Sports Medicine* (ACSM) cycling ergometry equation (ACSM, 2007). We opted for cycling over running as intervention mode for two reasons: (1) cycling on a stationary ergometer produces less head motion and whole body vibration and thus potentially prevents optode-scalp decoupling and (2) cycling exercise paradigms have shown more promise in enhancing post-exercise cognitive performance compared to running (Lambourne and Tomporowski, 2010; Zhu et al., 2021).

### 2.5 Familiarization

After fitness assessment, participants were familiarized with research facilities, experimental equipment, as well as study procedures. This was of particular importance to minimize learning effects in the n-back task both within and between sessions (Khaksari et al., 2019), and thereby essential to protocol feasibility. For the experiment, we used a custom PsychoPy script (v2023.2.2, Open Science Tools Ltd., Nottingham, UK) to present computerized numerical 1-, 2-, and 3-back tasks. During the familiarization session, participants completed a 15-min n-back training block, performed an 8-min cycling effort at their individualized moderate workload on the cycling ergometer Monark LC4 (Monark Exercise AB, Vansbro, Sweden) used in the main sessions, and had the fNIRS measurement set up.

### 2.6 Experimental procedure of the main sessions

**Figure 1:**
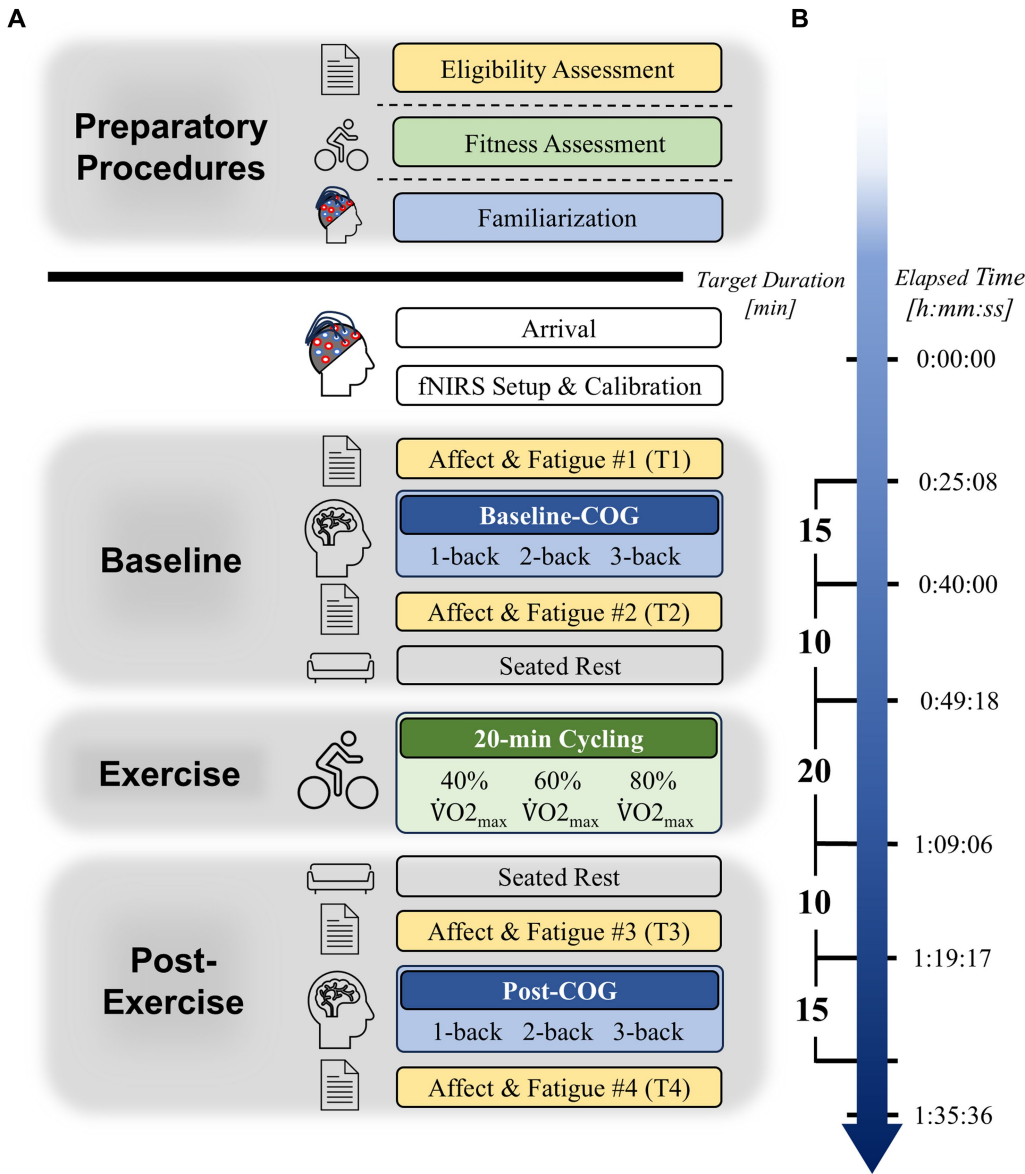
**(A)** Study overview. Three experimental sessions with randomized exercise intensities (40, 60, 80% VO2_max_) followed preparatory procedures. **(B)** Experimental protocol timeline for intervention sessions. Target durations (min, left) reflect the intended length of each protocol segment. Elapsed times (h:mm:ss, right) represent cumulative time from session start. Actual segment durations reported in the text were calculated between consecutive timepoints.

Figure 1A provides an overview of the study design and experimental timeline. Following arrival and fNIRS cap set-up, optode calibration was conducted via the Aurora fNIRS (v2021.9, NIRx Medizintechnik GmbH) in-built function.

#### 2.6.1 Working memory updating: n-back task and physiological measures

**Figure 2:**
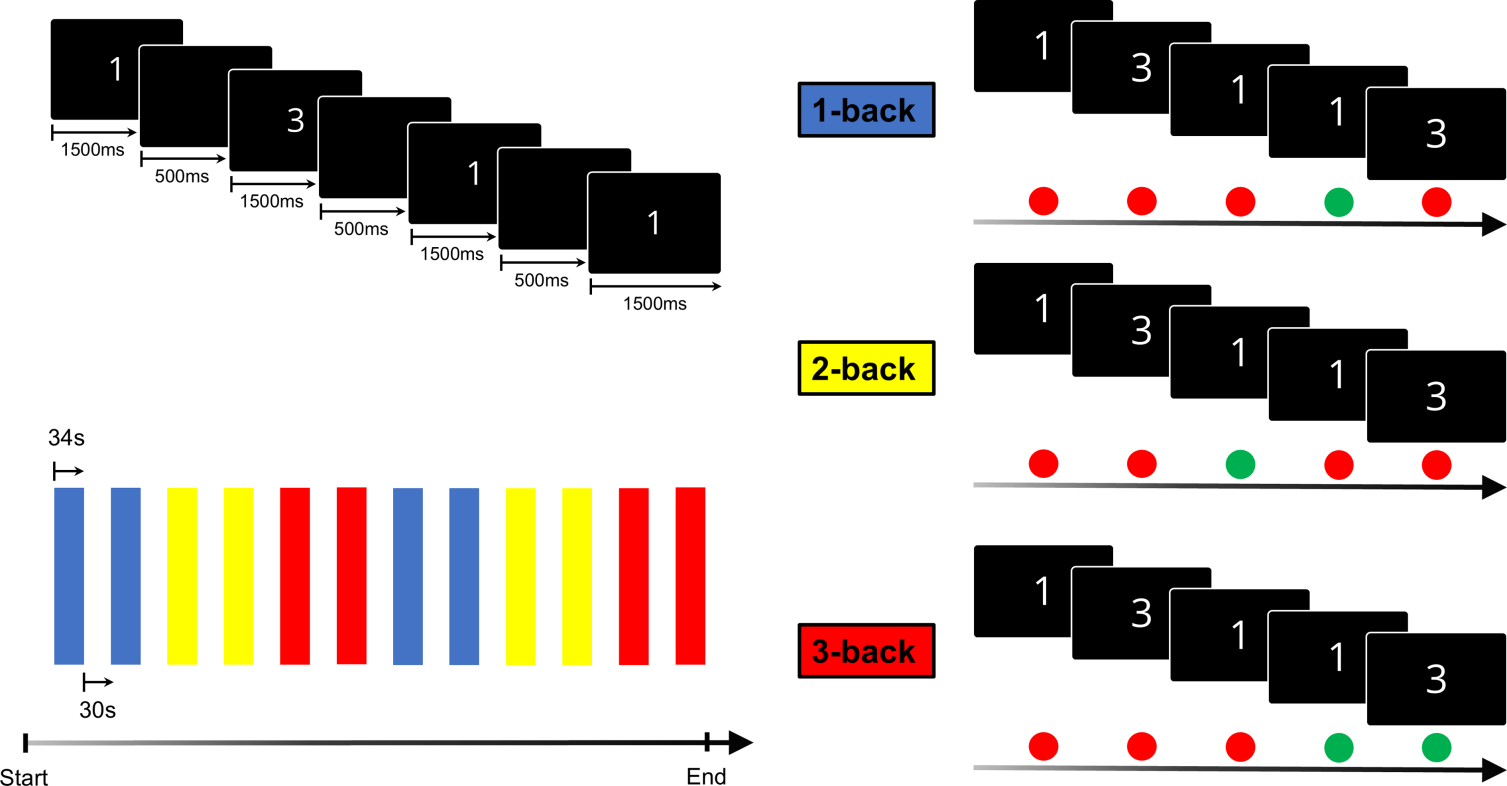
N-back task design. Trial structure with digit presentation duration (1,500 ms) and inter-stimulus interval (500 ms; upper left). Block sequence across the full task (1-1-2-2-3-3-1-1-2-2-3-3), with 34 s task blocks (colored) separated by 30 s counting baseline periods (white; lower left). Example target sequences for each difficulty level, where green dots indicate correct match responses (1-back: match with one position prior; 2-back: two positions prior; 3-back: three positions prior; right).

Subsequently, participants completed four blocks of 1-, 2-, and 3-back tasks in a fixed pattern (1-1-2-2-3-3-1-1-2-2-3-3), while seated in front of a Lenovo ThinkPad E15 Gen 3 20YG (Lenovo, Beijing, China). One of four pre-generated sequences of 17 digits (‘1’ to ‘9’, 7 targets and 10 foils) was randomly assigned to each experimental block and participants were instructed to indicate whether the presented digit matched the digit presented one (1-back), two (2-back), or three (3-back) steps before or not by pressing left and right arrow keys. Digits were presented for 1,500 ms with a 500 ms inter-stimulus interval, for a total block duration of 34 s. The response window was enabled for the full 2,000 ms before the next digit occurred. To limit mind-wandering (Herold et al., 2018) and preceding every block, participants were instructed to fixate at a white dot on the black screen and silently count upwards from ‘1’ for 30 s. Figure 2 illustrates the n-back setup.

Affect (Positive and Negative Affect Schedule, Mackinnon et al., 1999), and mental and physical fatigue (Visual Analogue Scale, Lee et al., 1991) were assessed immediately before and after each n-back task.

#### 2.6.2 Cortical hemodynamic response

**Figure 3:**
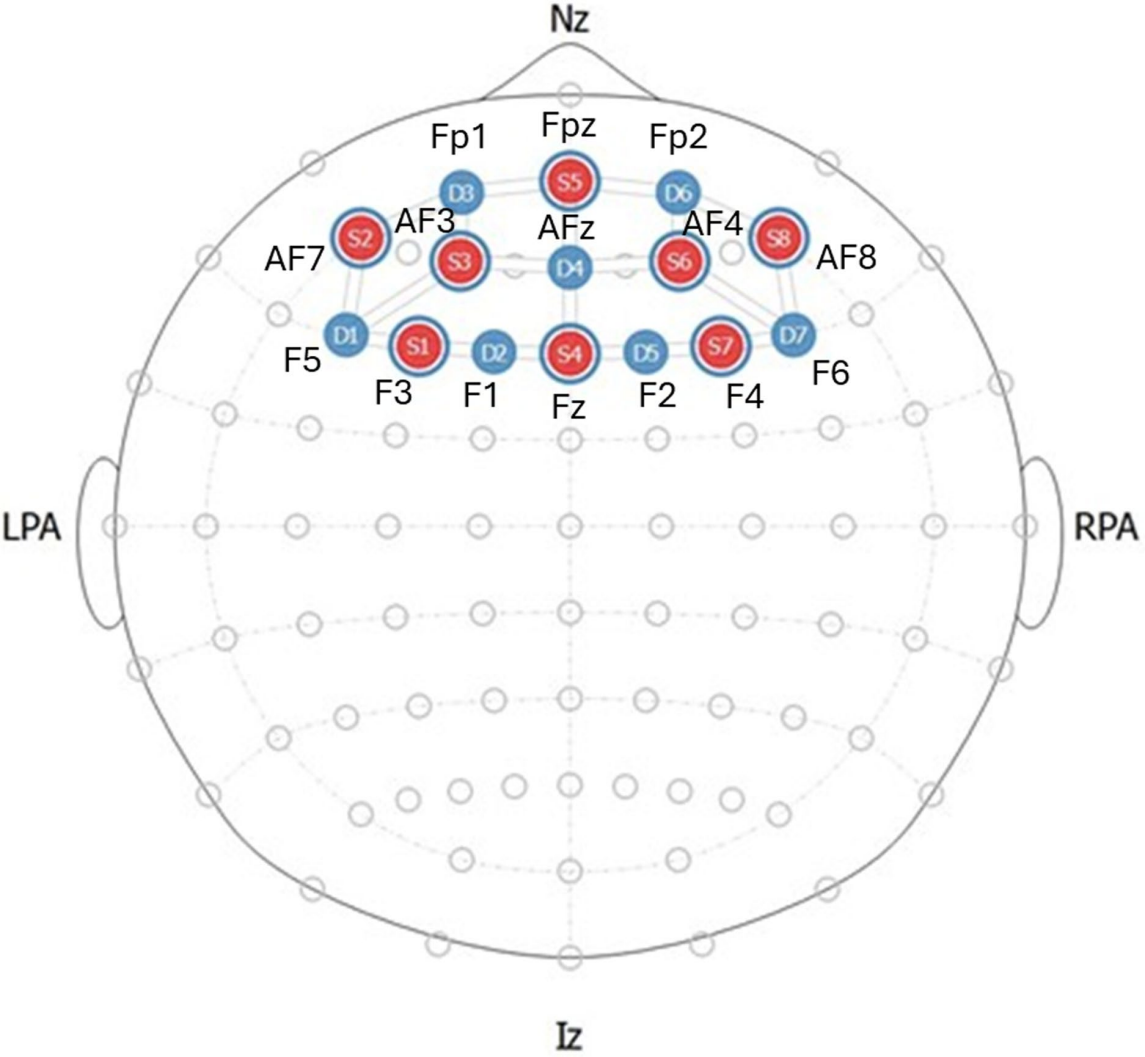
fNIRS optode montage. Twenty channels were built from eight source optodes (S, red) and seven detector optodes (D, blue) with 3 cm inter-optode distance. One detector optode was split into eight short-distance channels at 0.8 cm from each source optode, indicated by blue halos around each source optode. Optode placement adhered to the international 10–20 system and Fpz marked 10% of the distance from nasion to inion.

N-back task-induced changes in cortical oxygenation were measured utilizing a NIRSport2 (NIRx Medizintechnik GmbH, Berlin, Germany) continuous-wave fNIRS device with short-separation channels. Light was transmitted at wavelengths of 760 and 850 nm, and data was sampled at 10.17 Hz using Aurora fNIRS (v2021.9, NIRx Medizintechnik GmbH) recording software with predefined PFC montage (Figure 3). The montage layout consisted of eight illuminating sources and seven detector optodes with a separation distance of 3 cm. An additional detector optode was split into eight short-separation detectors placed at 0.8 cm from each source (Gagnon et al., 2012). Optode placement adhered to the international 10–20 system (Homan et al., 1987) over the PFC area on a 64-channel NIRScap (NIRx Medizintechnik GmbH), positioned midway between the pre-auricular points and the nasion and inion landmarks. The center of the Fpz emitting optode marked 10% of the distance between nasion and inion. The appropriate cap size (54, 56, 58, 60, or 62 cm) was selected based on the participant’s head circumference. No spatial registration was performed to verify optode placement. Instead, correct optode placement was ensured through standardized landmark-based markings on the head.

#### 2.6.3 Exercise intervention

The exercise intervention commenced with 1 minute of warm-up at 50 W. The resistance was then elevated to the target workload corresponding to 40, 60 and 80% of V̇O2_max_ for the remaining 19 min. The order of these work rates was counterbalanced and randomized between individuals. An experienced exercise physiologist accompanied all sessions to allow minor workload adjustments where necessary. Heart rate was continuously measured using a Polar Vantage M2 multisport watch (Polar Electro Oy, Kempele, Finland), and acute heart rate and perceived physical exertion (Borg, 1970) were acquired every 5 min during the intervention. Acute heart rate was later transformed into a percentage of maximum heart rate (Nes et al., 2013). The participants were imposed a 10-min seated rest before and after the intervention to prevent physiological and psychological cross-effects. Another block of 1-, 2- and 3-back tasks was administered after post-exercise rest.

### 2.7 Procedural feasibility assessment

Procedural feasibility was descriptively evaluated through participant attrition, protocol timing adherence, fNIRS setup reliability and target exercise load estimation. Additionally, affect and fatigue were examined as potential moderators, given that affective state (Pessoa, 2009) and mental fatigue (Van Der Linden et al., 2003) modulate prefrontal-dependent cognition. Protocol timing adherence was assessed by comparing actual task timing against the intended protocol. fNIRS setup reliability included live-quality rating generated by the Aurora software and recalibration rate. Load estimation success was evaluated by comparing individual *heart rates* (HR), *percentages of maximal heart rate* (%HRmax), and *ratings of perceived exertion* (RPE) against intensity-specific reference ranges set by the ACSM (Garber et al., 2011).

### 2.8 Data processing and scientific feasibility assessment

The raw fNIRS signal was preprocessed and analyzed using the MatLab-based (R2023b, MathWorks Inc., Natick, Massachusetts, United States) NIRS Brain AnalyzIR toolbox (Version 837, June 12, 2020, Santosa et al., 2018). For data quality assessment and channel rejection, multiple metrics were used independently. First, the SCI (Santosa et al., 2020) was calculated as the zero-lag cross-correlation between the cardiac-band signals of both wavelengths. Cortical component and other systemic components (i.e., respiration, Mayer-Waves) were attenuated using a band-pass filtering between 0.5 Hz and 2.5 Hz, corresponding to an expected heart rate range of 30–150 bpm (Hernandez and Pollonini, 2020). A high similarity of the cardiac component across both wavelengths indicates good optode-to-scalp coupling, with the SCI approaching a value of 1. Channels with an SCI lower than 0.8 were defined as having poor quality (Pollonini et al., 2016). SCI computation was carried out within the NIRS Brain AnalyzIR toolbox. An explorative *Quality-Testing of Near-Infrared Scans* (QT-NIRS) pruning sensitivity analysis was conducted within the NIRSplot toolbox^1^ (Hernandez and Pollonini, 2020). In QT-NIRS, channels are segmented into separate windows and evaluated based on SCI and PSP. PSP indirectly addresses motion by representing the ratio of cardiac-band peak power to cumulative power across all frequencies. It therefore functions as a sensible addition to SCI, as SCI is known to be inflated by motion artifacts (Pollonini et al., 2016). For the present analysis, channels were discarded if less than 75% of windows were ascribed good quality (Beaton et al., 2026). Quality thresholds were set to SCI ≥ 0.8 and PSP ≥ 0.1, and a 5-s window length was chosen (Hernandez and Pollonini, 2020). The proportion of discarded channels relative to the total number of channels was expressed as the pruning ratio.

Optical density was further transformed to changes in hemoglobin concentration using the modified Beer–Lambert law (Delpy et al., 1988) and a partial path-length factor (Toyoda et al., 2008) of 0.1, accounting for the portion of light propagating through actual brain tissue.

First-level analyses employed a *general linear model* (GLM) on the processed signal between each source-detector pair. A canonical hemodynamic response function template, peaking at 6 s after stimulus onset, was used to depict the response shape. An autoregressive iteratively reweighted least squares (AR-IRLS) pre-whitening filter (Barker et al., 2013) was applied, with nearest short-separation channels entered simultaneously as regressors into the GLM matrix to attenuate superficial physiological noise (Santosa et al., 2020). Short-channel regression was applied jointly to both *oxygenated hemoglobin* (oxyHb) and *deoxygenated hemoglobin* (deoxyHb) and slow signal drift was addressed through AR-IRLS pre-whitening rather than explicit drift regressors. Regression coefficients (*β*-values) for every channel, condition, task difficulty, and timing of the n-back task were derived from the GLM. The 30 s counting period before each n-back block served as baseline against which n-back task-related hemodynamic responses were modeled. SCI- and QT-NIRS-based channel pruning was conducted prior to GLM fitting. Agreement between *β*-estimates derived from each pruning approach was subsequently evaluated separately for oxyHb and deoxyHb using Lin’s concordance correlation coefficient (Lin’s CCC; Lin, 1989).

N-back task engagement was evaluated based on behavioral and prefrontal hemodynamic load effects, using only pre-exercise sessions to avoid confounding by exercise-induced effects on cognition. Reaction time averages were calculated from correct trials only, and both reaction time and accuracy were descriptively compared across task difficulty levels. For prefrontal load effects, SCI-based *β*-estimates from all three pre-exercise sessions were entered into a linear mixed model (LMM) with n-back difficulty as a fixed effect and a random intercept for participant [model formula: *β* ~ −1 + n-back difficulty + (1|subject)]. Post-hoc contrasts estimated the hemodynamic difference between 2-back and 1-back, and between 3-back and 1-back, with multiple comparisons corrected using the false discovery rate (FDR), with a *q*-value set at <0.05 indicating statistical significance. Channel-level *t*-statistics were averaged across the PFC to characterize overall significance of load effects.

## 3 Results

### 3.1 Study sample

After initial screening, 14 of 21 recruited participants met the study criteria. Exclusion criteria (Table 1) comprised above-threshold self-rated stress levels (5), above-threshold anxiety (3) and depression symptoms (1), and alcohol use disorder symptoms (2). A non-specified positive answer in the health declaration caused the final exclusion. Nine of the remaining 14 (*Mean (M)* age: 28.9 years, *SD* = 6.3, range: 21–38; *M* weight: 74.5 kg, *SD* = 11.7; *M* education: 15.1 years, *SD* = 1.6) completed the study and were included in the analyses. The final sample comprised seven males and two females with an average V̇O2_max_ of 53.3 mL·kg^−1^·min^−1^ (*SD* = 5.4).

### 3.2 Procedural feasibility

The protocol appeared largely executable, with technical and psychological burden remaining within descriptively acceptable ranges.

#### 3.2.1 Participant attrition

Among the five participants with incomplete data, two dropped out after the first experimental session for undisclosed reasons; two had missing fNIRS data from one or two sessions due to discomfort with the fNIRS cap, and one participant was excluded due to technical synchronization issues between PsychoPy and the NIRSport2 device. Headaches were the reason for both incomplete data sets related to physical discomfort. One participant wore the cap throughout the first two visits before requesting removal during the post-exercise rest period of the second visit. The third visit was completed without the cap. The other participant had the cap removed prior to cycling during the first visit but completed the two subsequent visits with the cap without complaints.

#### 3.2.2 Protocol timing adherence

A total of 27 experimental sessions were analyzed. Due to incomplete timestamp documentation across some sessions, protocol timing calculations were based on varying sample sizes (*n* = 13–21). The study protocol lasted an average of 95.6 min (*SD* = 7.1 min, *n* = 13). An average of 25.1 min (*SD* = 5.0 min, *n* = 21) elapsed before the start of the first n-back task. Seated rest durations before (*M* = 9.9 min, *SD* = 1.7 min, *n* = 18) and after (*M* = 10.0 min, *SD* = 0.4 min, *n* = 21) the cycling intervention closely matched the planned 10-min rest duration (Figure 1B).

#### 3.2.3 fNIRS setup reliability

Overall, fNIRS calibration was successful with only nine necessary recalibrations across 50 recordings. Documentation was missing in a further four cases. The sole reason requiring recalibration was bad or only acceptable optode coupling. After recalibration, no bad and three acceptable quality channels were used across all recordings. No adverse events for the n-back task were recorded.

#### 3.2.4 Target exercise load estimation

Average Ekblom-Bak-derived low-, moderate- and high-intensity workloads were 89 W (*SD* = 23.6), 129 W (*SD* = 30.6) and 169 W (*SD* = 44.8), respectively. Two individuals exhibited signs of exhaustion during the 8-min moderate-intensity familiarization, as calculated by the Ekblom-Bak test. Workload recalculations were therefore conducted for all participants; the adjusted values were 30% (*SD* = 14.5, range: 3.6–55.7) lower. Average heart rate during the last 15 min of the intervention was 112.0 bpm (*SD* = 7.2), 137.0 bpm (*SD* = 11.3) and 158.1 bpm (*SD* = 7.7) for low-, moderate- and high-intensity conditions. %HR_max_ and RPE ratings matched targeted exertion. Table 2 depicts outcomes related to exercise load estimation.

**Table 2:** Intervention outcomes (heart rate – HR; percentage of maximal heart rate – %HR_max_; rating of perceived exertion – RPE) indicates successful workload control across intensity conditions.

Outcome	Intervention		
	HR	%HR_max_	RPE
Condition	Mean (SD)	Mean (SD)	Mean (SD)
Low	112.0 (7.2)	58.2 (3.5)	11.0 (1.5)
Moderate	137.0 (11.3)	71.1 (5.2)	13.6 (1.5)
High	158.1 (7.7)	82.1 (3.8)	16.3 (0.5)

Values are Mean (SD).

#### 3.2.5 Affect and fatigue

**Figure 4:**
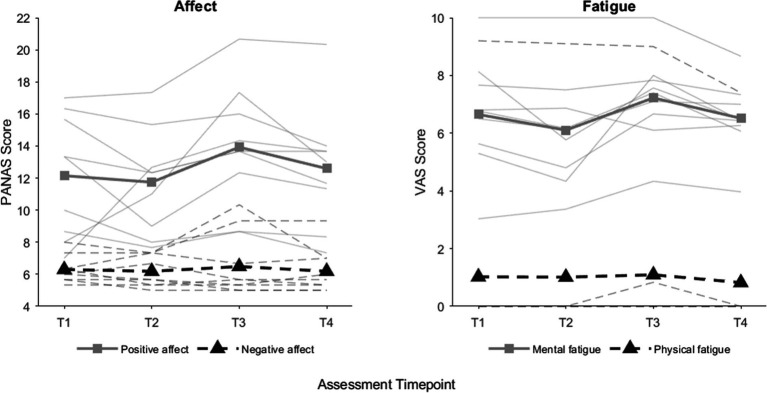
Development of affect (left) and fatigue (right) over timepoints, averaged across exercise intensity conditions (mean and individual trajectories). Affect was rated on the Positive and Negative Affect Schedule (PANAS); fatigue was indicated on a Visual Analogue Scale (VAS, 0–10). Higher values indicate greater affect and perceived fatigue. T1 = before baseline n-back task; T2 = after baseline n-back task; T3 = before post-exercise n-back task; T4 = after post-exercise n-back task. Near-uniform physical fatigue reflects a floor effect, with 88% of responses at zero.

Participants’ self-reported affect and fatigue remained stable across time points and conditions (Figure 4; Table 3). Average positive affect ranged from 11.7 (*SD* = 3.2) to 13.9 (*SD* = 3.9) across timepoints, and average negative affect from 6.2 (*SD* = 1.4) to 6.5 (*SD* = 2.0), indicating consistently moderate positive and low negative affect throughout the protocol. Average mental fatigue was similarly stable, ranging from 6.1 (*SD* = 1.5) to 7.2 (*SD* = 1.9), with a slight peak at T3 following the exercise intervention. Physical fatigue was minimal in most participants, with 88% of responses at zero (*M* range: 0.8–1.1).

**Table 3:** Affect and fatigue showed stability across timepoints, with positive affect (PANAS+, range 5–25), negative affect (PANAS-, range 5–25), mental fatigue (Visual Analogue Scale, 0–10), and physical fatigue (Visual Analogue Scale, 0–10) remaining largely unchanged.

Timepoint	Affect and fatigue			
	T1	T2	T3	T4
Outcome	Mean (SD)	Mean (SD)	Mean (SD)	Mean (SD)
PANAS+	12.1 (3.8)	11.7 (3.2)	13.9 (3.9)	12.6 (3.8)
PANAS−	6.3 (0.9)	6.2 (1.0)	6.5 (2.0)	6.2 (1.4)
Mental fatigue	6.6 (2.0)	6.1 (1.9)	7.2 (1.5)	6.5 (1.2)
Physical fatigue	1.0 (3.1)	1.0 (3.0)	1.1 (3.0)	0.8 (2.5)

T1 = before baseline n-back task; T2 = after baseline n-back task; T3 = before post-exercise n-back task; T4 = after post-exercise n-back task. Values are Mean (SD).

### 3.3 Scientific feasibility

High data quality across condition, near-perfect concordance between SCI- and QT-NIRS-derived *β*-estimates, and n-back sensitivity to cognitive load in behavioral and hemodynamic prefrontal domains suggest scientific feasibility under these protocol conditions.

#### 3.3.1 Data quality and metric concordance

SCI computation yielded an average baseline (pre-exercise) SCI of 0.964 (*SE =* 0.003). Post-exercise SCI values were slightly lower for low- (*M* = 0.961, *SE* = 0.006) and moderate- (*M* = 0.958, *SE* = 0.005) intensity conditions and slightly higher for the high- (*M =* 0.975, *SE* = 0.004) intensity condition.

**Figure 5:**
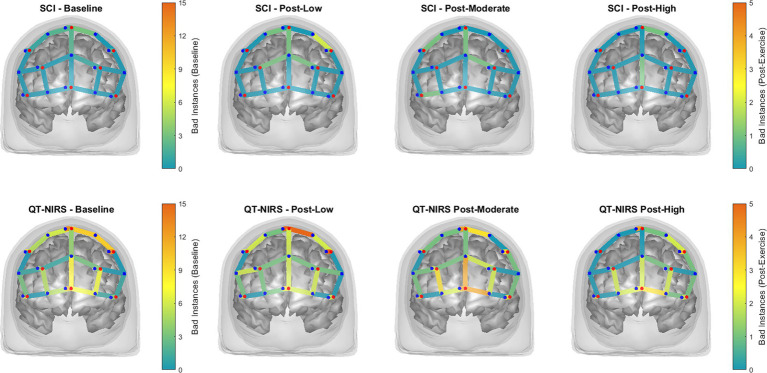
Spatial distribution of excluded channels using SCI (upper row) versus QT-NIRS (lower row) pruning methods during baseline and post-exercise n-back tasks. Low, moderate and high intensities correspond to workloads at 40, 60, 80% VO2_max_. Color scale indicates the number of excluded channels per location. Channels were flagged based on SCI, if cross-correlation between wavelengths did not reach 0.8. QT-NIRS flagged channels, if less than 75% of five-second windows passed quality threshold of SCI ≥ 0.8 and PSP ≥ 0.1. QT-NIRS identified substantially more poor-quality channels than SCI alone, particularly in lateral and cranial prefrontal regions.

SCI-based pruning was stable across conditions, highest pruning ratio was observed after low-intensity cycling (2.8% of channels), lowest pruning ratio after high-intensity cycling (0.4% of channels). In total, 2.1% of channels failed to meet the SCI threshold of 0.8 (Figure 5).

**Figure 6:**
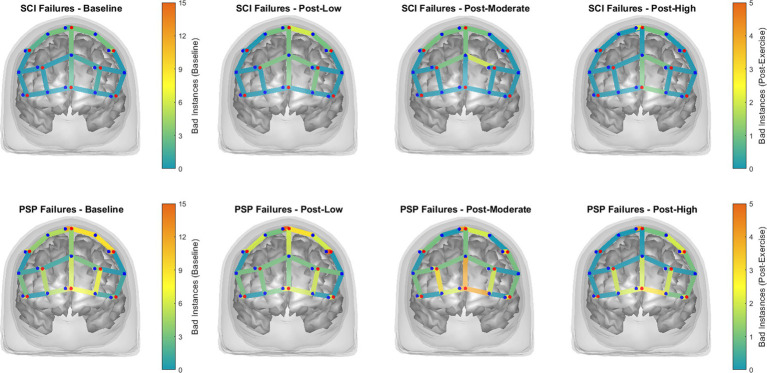
Decomposition of QT-NIRS channel exclusions by failure criterion: SCI failures (upper row) versus PSP failures (lower row) during baseline and post-exercise n-back tasks. Low, moderate and high intensities correspond to workloads at 40, 60, 80% VO2_max_. Color scale indicates the number of excluded channels per location. Channels were flagged for SCI failure if fewer than 75% of five-second windows met an SCI threshold of ≥0.8, and for PSP failure if fewer than 75% of windows met a PSP threshold of ≥0.1. The majority of QT-NIRS exclusions resulted from insufficient peak spectral power rather than poor scalp coupling, with no consistent spatial failure across conditions.

**Figure 7:**
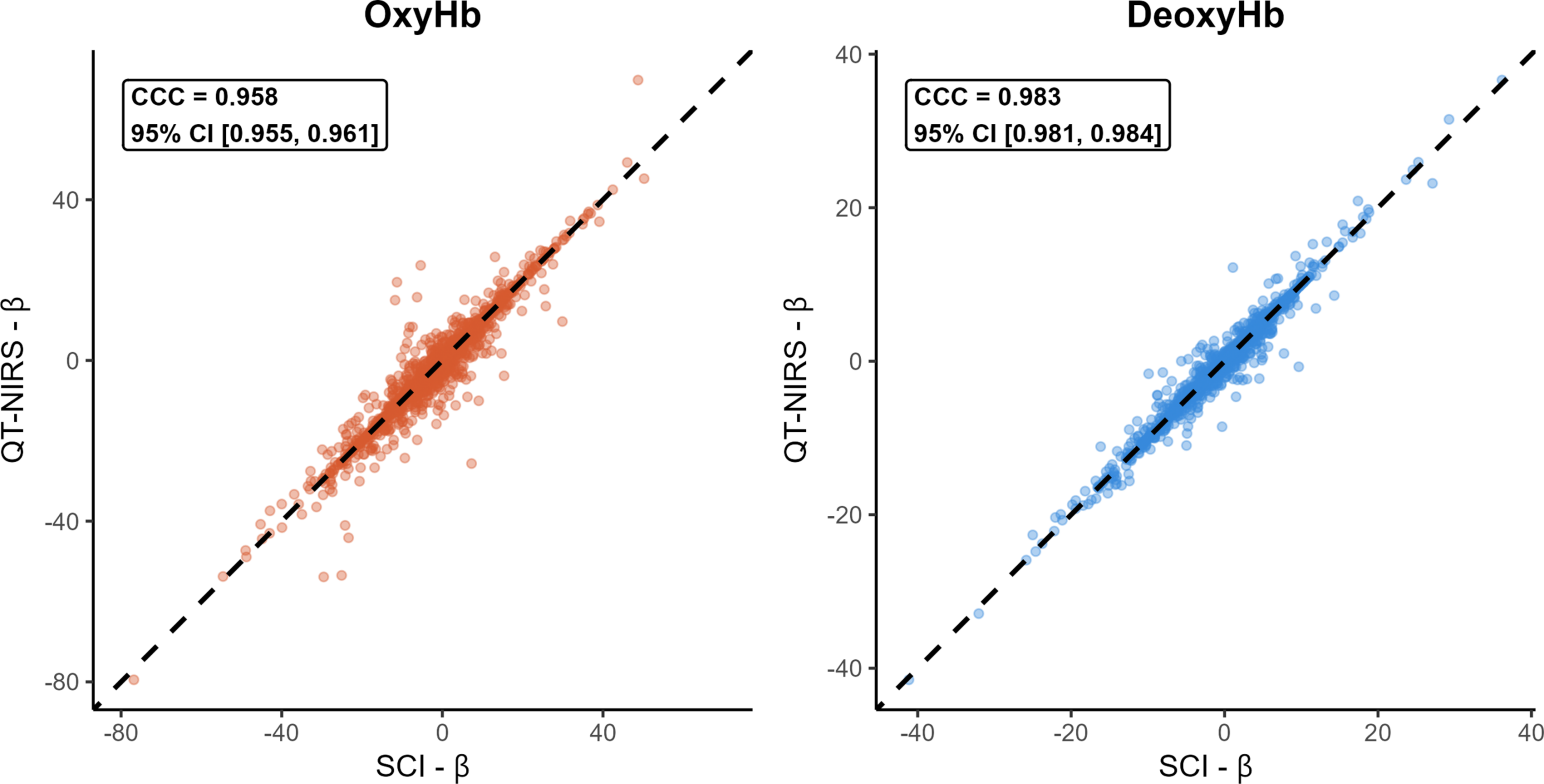
Concordance between SCI and QT-NIRS pruning methods. High Lin’s CCC between *β*-estimates from both pruning approaches for oxyHb (0.958) and deoxyHb (0.983).

Pruning stability was also observed using the QT-NIRS approach. The highest pruning ratio was observed after moderate-intensity cycling (19.8%), while the lowest was observed after high-intensity cycling (14.3%). Across all timepoints and conditions, 18.2% of channels were discarded via QT-NIRS pruning (Figure 5). In QT-NIRS, 216 channels were discarded based on insufficient PSP, 15 based on low SCI, and a further 44 based on both criteria (Figure 6). Visual inspection indicates SCI failures predominantly in cranial and central regions, whereas PSP failures occurred without any clear spatial pattern. Concordance correlations of SCI- and QT-NIRS-derived *β*-estimates were *CCC* = 0.958 (95% *CI* [0.955, 0.961]) for oxyHb and *CCC* = 0.983 (95% *CI* [0.981, 0.984]) for deoxyHb (Figure 7).

#### 3.3.2 N-back task engagement

**Figure 8:**
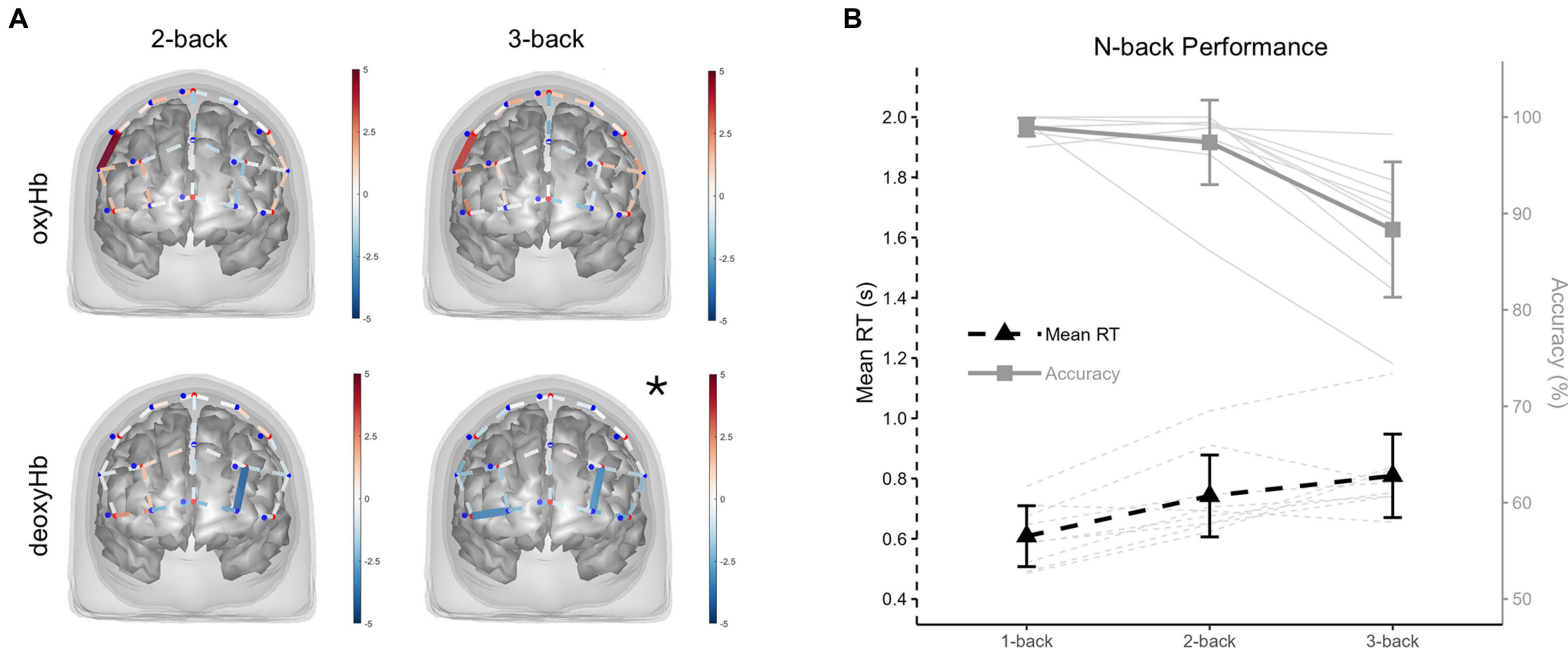
**(A)** Hemodynamic load effects. Prefrontal cortex oxyHb (top) and deoxyHb (bottom) β-estimates for 2-back (left) and 3-back (right) contrasted against 1-back, averaged across 27 pre-exercise sessions (9 participants). Red indicates positive β-estimates, blue negative. Thick solid channels mark significance on the channel-level. Asterisks denote whole-PFC significance of contrasts after FDR correction (*q* < 0.05). **(B)** Behavioral performance across n-back difficulty levels with individual participant trajectories; Group Mean ± SD displayed as dashed black line with triangles (Reaction Time) and solid grey line with squares (Accuracy).

Fixed effects of n-back difficulty on prefrontal hemodynamics were observed across all 27 pre-exercise recordings, pointing toward increased cognitive load with higher task difficulty (Figure 8A). In the 2-back to 1-back contrast, neither oxyHb (*β* = 0.60, *SE* = 0.49, *t*(72) = 1.24, *q* = 0.22) nor deoxyHb (*β* = −0.45, *SE* = 0.22, *t*(72) = −2.04, *q* = 0.09) reached significance after FDR correction. The 3-back to 1-back contrast, however, revealed a significant effect for deoxyHb (*β* = −1.16, *SE* = 0.23, *t*(72) = −5.13, *q* < 0.001), while oxyHb increased (*β* = 0.97, *SE* = 0.50, *t*(72) = 1.95, *q* = 0.055), but narrowly missed significance.

Across all 27 pre-exercise recordings, reaction time increased progressively from 1-back (*M* = 0.61 s, *SD* = 0.10) to 2-back (*M* = 0.74 s, *SD* = 0.14) and 3-back (*M* = 0.81 s, *SD* = 0.14), indicating increasing cognitive load. Accuracy remained high across 1-back (*M* = 99.0%, *SD* = 0.9) and 2-back (*M* = 97.4%, *SD* = 4.4) conditions, with a more pronounced decline at 3-back (*M* = 88.3%, *SD* = 7.0). The descriptive pattern of increasing reaction time and decreasing accuracy with higher task difficulty (Figure 8B) is consistent with the expected n-back load effect.

## 4 Discussion

In the present study, we investigated the feasibility of using fNIRS to monitor changes in n-back task-induced PFC activation following low-, moderate-, and high-intensity continuous cycling exercise in young, healthy adults. Procedural feasibility was evaluated based on participant attrition, protocol timing adherence, fNIRS setup reliability, target exercise load estimation, and changes in affect and fatigue. Scientific feasibility was assessed via signal quality and *β*-concordance of SCI- and QT-NIRS-based pruning.

### 4.1 Attrition, temporal precision and fNIRS setup reliability

The study protocol was largely executable. Two of 14 participants withdrew from the study after the preparatory session. A further three were discontinued after study enrollment: one due to technical issues with trigger events and two due to discomfort from fNIRS cap wear during one and two experimental sessions. As this pilot study was specifically designed to identify protocol-specific challenges, attrition was anticipated. Attrition in complex within-subject crossover studies can be costly both personally and financially; anticipating likely loci of dropout is therefore of practical significance to researchers. Preparation for technical failures and participant withdrawal can be as effective in limiting cost as the use of analytical methods robust to missing data points. In the present study, attrition predominantly stemmed from equipment-related factors rather than unforeseen withdrawals, suggesting that technical refinements could improve future retention.

Actualized section durations generally matched targeted timings. Consistent post-exercise rest is critical, as non-cortical hemodynamics such as heart rate and skin blood flow can remain elevated for up to 8 min after exercise cessation (Byun et al., 2014a), and meta-analytic evidence suggests that the largest cognition-enhancing effects occur 10 to 20 min post-exercise (Chang et al., 2012). Average post-exercise rest durations of 10.0 min (*SD* = 0.4 min, range: 9–11) across all sessions indicate well-standardized rest and further suggest that exercise-induced heart rate elevations had largely normalized before cognitive testing. Although data availability was limited due to incomplete timing documentation, successful adherence to the post-intervention protocol indicates the general applicability of the setup. The larger variability of rest before intervention (*M* = 9.9 min, *SD* = 1.7 min, range: 8–15 min) indicates greater precision is required in manual documentation or protocol adherence, but likely was not detrimental to analyses in this study. Taken together with the predictable and low-burden fNIRS setup – underscored by only nine recalibrations across 50 recordings and no poor-quality channels remaining after calibration – the present results suggest that researchers can reliably execute protocols with high demand on temporal precision.

### 4.2 Exercise intensity and fitness assessment

We prescribed cycling at 40, 60 and 80% of individual V̇O2_max_, adhering to ACSM guidelines for low, moderate and high physical loads (Garber et al., 2011). Initial load estimations using the Ekblom-Bak test proved problematic, as participants showed signs of exhaustion in both moderate-, and high-intensity conditions. The Ekblom-Bak test is known to overestimate fitness levels, particularly in low-fit populations (Björkman et al., 2016). Although our sample’s estimated V̇O2_max_ (*M* = 53.3 mL·kg^−1^·min^−1^, *SD* = 5.43), suggested well-above-average fitness, the observed exercise intolerance indicated systematic overestimations. Workloads were subsequently adjusted using the ACSM cycling ergometry equation (ACSM, 2007) and individualized estimation. After adjustment, acute heart rate remained within the prescribed target values throughout the session. The mean heart rate (expressed as a percentage of maximal heart rate) and perceived exertion during the 15 final minutes of each condition were within the threshold established by the ACSM, except for RPE during moderate-intensity cycling. This mismatch may be attributable to low sample size, subjective misestimation and multiple metric observation and could therefore reflect a random occurrence. Globally, load metrics fell well within the ACSM threshold, supporting successful load adjustment. Nevertheless, we recommend alternative fitness estimation practices in further investigations. For healthy populations, all-out testing is advised due to its relative safety (Goodman et al., 2011) and superior performance in V̇O2_max_ estimation (Jackson and Ross, 1996), ideally conducted in consultation with a medical practitioner (Riebe et al., 2015). Workloads for target intensity levels should be estimated using the ACSM leg ergometry equation, though experiential load adjustment remains advisable given potential misestimation (Lang et al., 1992; Koutlianos et al., 2013).

### 4.3 Psychological confounds

Both positive and negative affect remained stable across all timepoints and exercise intensities, indicating that the protocol did not induce affective responses that threaten interpretability. This stability is methodologically important, as negative affect has been shown to impair cognitive performance (Brose et al., 2012) and psychological stress is related to alterations of PFC oxygenation (Wang et al., 2005), with effects persisting beyond the stressor itself. Similarly, acute emotional stimulation can induce substantial changes in prefrontal hemodynamics as measured by fNIRS (Matsukawa et al., 2018), potentially introducing unwanted variance in cerebral oxygenation measurements.

Our findings on mental fatigue further underline this perspective. Mental fatigue peaked immediately following the exercise intervention and was lowest after n-back task segments, with values returning to near-baseline by protocol completion. This pattern suggests that the n-back tasks may have had an engaging or activating effect on participants, rather than inducing fatigue. This observation aligns with research showing that acute cognitive engagement can enhance alertness (Warm et al., 2008), whereas intense physical exercise has been shown to transiently increase subjective feelings of mental fatigue (Meeusen et al., 2021). Importantly, the transient nature of this effect, with fatigue returning to baseline within the protocol-timeframe, provides preliminary support for the feasibility of our experimental design for repeated cognitive assessment following exercise at varying intensities.

### 4.4 Scientific feasibility: SCI and QT-NIRS

The average SCI for all channels approached the maximum value of 1 (0.964, *SE* = 0.003), with only 2.1% of channels failing to reach the threshold of 0.8 (Pollonini et al., 2016) for sufficient data quality. Furthermore, no declines in SCI values were observed from baseline to post-exercise. In fact, fewer channels were flagged and pruned for insufficient scalp coupling after moderate- and high-intensity conditions, which may be attributable to moisture from perspiration enhancing optode-to-scalp coupling. These findings align with those of Kvist et al. (2023), who obtained similar SCI values in single- and dual-task walking protocols. Thus, light head motion and whole-body vibration do not appear to interrupt scalp coupling, suggesting that, with proper cap fitting and optode placement, fNIRS is a feasible tool to monitor PFC oxygenation changes even when exposed to mild movement or perspiration induced by cycling on a stationary bicycle ergometer.

Although SCI has been established as a robust metric for evaluating signal quality (Santosa et al., 2020), recent work (Ayaz et al., 2022; Yücel et al., 2025) has highlighted the QT-NIRS approach as potentially refining quality screening. To our knowledge, no study has directly compared SCI-only pruning to QT-NIRS pruning. Beaton et al. (2026) demonstrated that QT-NIRS pruning maintains higher signal-to-noise ratios in comparison to coefficient of variation-based pruning, indicating that multi-component QT-NIRS may be superior for channel rejection. Therefore, we conducted an explorative sensitivity analysis comparing both approaches.

Conservative SCI pruning retained 97.8% of channels, whereas aggressive QT-NIRS pruning only preserved 81.2% of channels. Despite this discrepancy, cross-method concordance was remarkably high for both chromophores (oxyHb *CCC* ≈ 0.96; deoxyHb *CCC* ≈ 0.98), supporting robust neural signal extraction and high data quality at the channel level. The slightly higher agreement of deoxyHb is consistent with its known robustness to extracerebral contamination (Kirilina et al., 2012; Tachtsidis and Scholkmann, 2016). The observed cross-method concordance may indicate that low levels of motion and exercise-induced optode-to-scalp coupling disruptions were insufficient to hamper signal quality when assessed via the SCI. This suggests that the SCI alone may be a sufficient pruning method where motion is limited and coupling disruptions are minimal. Whether QT-NIRS offers meaningful advantages over SCI in higher-motion situations remains an open question to be addressed by adequate experimental setups.

### 4.5 N-back task engagement

The n-back task produced load-dependent effects at both behavioral and prefrontal hemodynamic levels. Descriptively, reaction time increased and accuracy decreased with higher cognitive load across all 27 pre-exercise recordings, consistent with previously reported load-dependent effects in the n-back task among young adults (Yeung and Han, 2023). Prefrontal hemodynamics followed the same directional trend, with a spatially coherent pattern of decreased deoxyHb across the PFC in the 3-back to 1-back contrast. OxyHb increased, although not statistically significant. The 2-back to 1-back contrast did not reach statistical significance for both chromophores. This is consistent with an fMRI study by Lamichhane et al. (2020), who observed n-back-related PFC activity increases up to 3-back, with n-back loads exceeding 3-back leading to progressive PFC disengagement. Theories explaining this so-called inverted-U shape between cognitive loads and PFC activation include peaking WM capacity at the inflection point (Callicott, 1999) or task disengagement (Jaeggi et al., 2007). Another study by An et al. (2025) observed this inflection point at 2-back, when fNIRS was used to monitor PFC activation during an alphabetical n-back task. Although available evidence is heterogeneous, a clear pattern emerges and our findings preliminarily support this: Cognitive loads greater than 1-back induce elevated PFC activation.

Our choice to use a silent counting period as the inter-block baseline may have introduced unwanted PFC activation, thereby reducing the contrast between baseline and n-back activation. Herold et al. (2018) reported that mind-wandering – the dispersion of attention away from the target locus – may be a confound fNIRS is sensitive to (Durantin et al., 2015). The authors further raise the idea that simple cognitive tasks, such as counting (Holtzer et al., 2015, 2016), may be suitable to minimize disadvantageous effects of mind wandering by imposing cognitive engagement that is sub-threshold to PFC recruitment. Although promising, empirical evidence to support this is limited. Since counting recruits verbal, attentional and executive processes (Logie and Baddeley, 1987; Diamond, 2013), all of which are associated with PFC engagement (Diamond, 2013; Hu et al., 2019), it is plausible that the counting task may have induced PFC activation rather than minimizing it. Under these circumstances, the calculated contrasts may be conservative and thus underestimate the load effect (e.g., higher contrast is needed to reach significance).

Taken together and in light of heterogeneous n-back load-activation literature (Lamichhane et al., 2020; Meidenbauer et al., 2021; An et al., 2025), the observed activation gradient from 1-back to 3-back should be cautiously interpreted as evidence for n-back task sensitivity of PFC activation. Conservative activation estimation, resulting from potential underestimation of load effects due to unsuccessful PFC disengagement during baseline blocks could further sharpen the picture toward distinct load effects. Adequately powered studies and validated PFC disengagement during baseline blocks are needed to further strengthen these preliminary feasibility indications.

## 5 Strengths, limitations and future directions

The within-subject crossover design, inclusion of short-separation channels, and application of contemporary preprocessing pipelines represent methodological strengths that support the internal validity of procedural and signal quality findings.

This study is not without limitations. The sample size of nine participants precludes inferential conclusions across outcomes. Pooling data across three experimental sessions per participant increases inferential robustness, though this can introduce systematic individual effects, thus the pooling benefit may be partially offset. Results should therefore be interpreted as exploratory rather than confirmatory. Due to the large number of channels included in the data quality assessment, signal quality outcomes are comparatively more robust.

Optode registration via spatial digitization was not conducted in this study to limit experiment duration and thus participant burden, consistent with the procedural feasibility objectives. Optode placement was standardized via markings on the participants’ head. As analyses were restricted to the whole PFC as region of interest, precision could still be achieved without procedural feasibility being compromised. Future studies adopting spatial registration should account for increased setup time when protocols are designed.

A fixed n-back block order was chosen over a counterbalanced design, as the latter was considered likely to introduce confusion and fatigue, stemming from cycling through three load levels. However, using a fixed block order may have instead introduced confounds related to time-on-task, fatigue, expectation or hemodynamic carryover. The limited pool of four pre-generated digit sequences increases the likelihood of sequence familiarity, which could alter hemodynamic responses and performance across repeated sessions. Larger sequence pools could mitigate systematic effects and counterbalanced block orders should be considered, though possibly introducing unwanted effects in multi-load paradigms. Additionally, the use of a silent counting period as the inter-block baseline represents a methodological limitation, as counting may recruit verbal, attentional, and executive processes and thereby introduce PFC activation into the baseline condition – lowering cognitive load effect contrasts. Future works should prioritize baseline conditions with empirical support for PFC disengagement to enable more accurate detection of task-related activation.

Incomplete timestamp documentation limits the interpretability of procedural feasibility findings; future feasibility studies and experimental trials should more rigorously document relevant events to ensure transparency and inform study designs. Additionally, acquisition of participant characteristics known to alter fNIRS signals (e.g., hair architecture and skin color) could have aided in tracing bad signal quality sources and thereby added nuance to the interpretation of bad quality channels.

While our results suggest QT-NIRS as a potentially viable pruning technique, further research should examine the effectiveness of QT-NIRS pruning in comparison to SCI-based channel rejection and its effect on overall channel quality.

## 6 Conclusion

In this study, we investigated the feasibility of a three-arm within-subject study protocol using fNIRS to capture n-back task-induced PFC oxygenation changes after continuous cycling at different intensities in young and healthy adults. Scientific feasibility was evaluated through dual-metric data quality assessment (SCI and QT-NIRS) and n-back task sensitivity. Procedural feasibility was assessed via attrition, protocol timing adherence, fNIRS setup reliability, target exercise load control, and monitoring of affect and fatigue. Our results provide preliminary support for the scientific feasibility of the experimental setup, indicated by high data quality and probable n-back task sensitivity. Successful protocol timing adherence and stable affect and fatigue point toward procedural feasibility, with refinements warranted to fitness assessment and participant enrollment. These findings suggest that with rigorous quality control, fNIRS could reliably capture PFC activation in study protocols lasting up to 90 min and when light motion is present. This provides a foundation for investigating the neural mechanisms underlying exercise-induced cognitive benefits.

## Data Availability

The raw data supporting the conclusions of this article will be made available by the authors, without undue reservation.

## Footnotes

1 Available at: https://github.com/lpollonini/qt-nirs, downloaded on: December 04, 2025.
